# Neutrophil gelatinase-associated lipocal in protein levels as an acute appendicitis biomarker in children

**DOI:** 10.1186/s40064-016-1853-x

**Published:** 2016-02-27

**Authors:** Unal Bakal, Mehmet Saraç, Harun Ciftci, Tugay Tartar, Esra Kocdemir, Suleyman Aydin, Ahmet Kazez

**Affiliations:** Department of Pediatric Surgery, School of Medicine, Firat University, 23119 Elazig, Turkey; Department of Medical Biochemistry, Ahievran University, 4000 Kirsehir, Turkey; Kovancilar State Hospital, 23850 Elazig, Turkey; Department of Medical Biochemistry and Clinical Biochemistry (Firat Hormones Research Groups), School of Medicine, Firat University, 23119 Elazig, Turkey

**Keywords:** Lipocalin, Acute appendicitis, Biomarker, Pediatric surgeon

## Abstract

**Objective:**

Appendicitis is very commonly encountered in emergency clinics. There is an urgent need for early and accurate predictive biomarkers of appendicitis in order to save lives, because currently-available biomarkers are imprecise and their delayed response impairs the ability of emergency doctors and pediatric surgeons to provide timely and potentially effective therapies. This study was performed to determine whether changes in the blood levels of neutrophil gelatinase-associated lipocalin (NGAL) can help to diagnose acute appendicitis in children and distinguish acute appendicitis from abdominal pain.

**Methods:**

Sixty children were enrolled and divided into three groups, with 20 patients per group: Group 1 (patients with appendicitis), Group 2 (patients with abdominal pain) and Group 3 (control). Blood NGAL levels were determined by ELISA.

**Results:**

The basal average serum NGAL levels were 8.2 ng/ml for Group 1, 3.9 ng/ml for Group 2, and 3.3 ng/ml for Group 3. Twenty-four and 72 h after surgery the levels were 5.1 and 2.8 ng/ml, respectively, in Group 1, 2.9 and 2.8 ng/ml in Group 2, and 2.6, 2.7 ng/ml in Group 3. Setting the cut-off point to 7 generated an area under the receiving operating curve (ROC) curve at 95 % confidence interval with 77.3 % sensitivity and 97.4 % specificity.

**Conclusion:**

These data indicate a significant difference in NGAL values between basal and postoperative measurements in appendicitis patients (p < 0.05). The ROC curve results showed that NGAL is a promising novel biomarker for the differential diagnosis of acute appendicitis from abdominal pain.

## Background

Appendectomy is the most commonly-performed emergency operation in pediatric surgery clinics. The clinical signs of appendicitis arise from an inflammatory process that begins as a luminal obstruction and proceeds to abdominal pain. Diagnosis is based on clinical findings and there is still no definitive laboratory diagnosis. Clinical signs of appendicitis can be confused with many diseases associated with abdominal pain (Alvarado 1986; Apak et al. 2005; Brănescu et al. 2012; Nance et al. 2000; Rodriguez-Sanjuan et al. 1999).

Early detection of appendicitis is crucial, as prompt treatment leads to better patient outcomes and fewer complications. Currently, the Alvarado scoring system is used in the diagnosis of acute appendicitis and is calculated out of a total of 10 points on the basis of symptoms, clinical findings, leukocyte count and radiological findings. However, the negative laparotomy rate is low when the score on this system is greater than 7. Observation and/or additional definite laboratory evaluation are needed for scores of 5–6 (Alvarado 1986). Clinicians still encounter problems such as perforation, negative appendectomy, mortality, morbidity, and long hospital stays in patients with acute appendicitis (Flum and Koepsell 2002).

We hypothesized that there could be a link between appendicitis and neutrophil gelatinase-associated lipocalin (NGAL) = lipocalin 2 (LCN2) = siderocalin because appendicitis is inflammation of the appendix: the inflamed appendix becomes infected with intestinal bacteria. Neutrophils are the primary white blood cells that respond to a bacterial infection. NGAL is mainly expressed by neutrophils and at low levels by the kidney, prostate, and epithelia of the respiratory and alimentary tracts and appendix (Bartsch et al. 1995; Cowland and Borregaard 1997; Leelawat et al. 2011; Nielsen et al. 1996). Furthermore, recent evidence suggests that NGAL could be involved in the pathophysiology of chronic renal diseases such as polycystic kidney disease and glomerulonephritis. It also appears to be up regulated in cells under “stress”, e.g. from infection, inflammation, ischemia or neoplastic transformation. If the infected appendix causes an increment of neutrophils, more NGAL will be secreted into the blood stream. Theoretically, therefore, increased plasma NGAL might be an indicator of acute appendicitis. The purpose of this study was to assess whether serum NGAL levels could be useful for diagnosing acute appendicitis and could distinguish acute appendicitis from abdominal pain.

## Methods

The Clinical Research Ethics Committee of Firat University Faculty of Medicine approved our study (date: 02/23/2012, session #7). A total of 60 children who were treated in the Department of Pediatric Surgery from December 2012 to 2014 were included in our study. The patients were divided into three groups of 20 each. Alvarado scores were noted for each subject. Patients were not separated on the basis of age or gender. Parents of all patients in the study were informed prior to the study and informed consent was obtained from them. The age and cognitive skills of the children prevented them from using a visual analog scale to evaluate their appetite, so their parents stated whether the children had a normal appetite or loss of appetite. Patients with congenital anomalies and/or life-threatening conditions and patients who had had an appendectomy were excluded from the study. The groups were divided as follows: Group 1: patients diagnosed with appendicitis due to undergo appendectomy; Group 2: patients with abdominal pain but no appendicitis; Group 3: controls (healthy children who had tested negative for cryptorchidism, inguinal hernia, phimosis and vulvar fusion at the pediatric surgery department, and had no known diseases). Blood samples were obtained at time zero (preoperative, basal), 24 h postoperative and 72 h postoperative in Group 1. Blood samples were also obtained from Groups 2 and 3 at the corresponding times by a previously published method (Aydin 2015). Patients with acute appendicitis are usually admitted to our hospital in the afternoon; thus, blood samples in Groups 2 and 3 were obtained during the same time period (at about 3:00 pm) to avoid any effects of circadian rhythm. The samples were stored at −80 °C pending analysis. The WBC count and neutrophil percentage were measured with an ABX Pentra DX SPS Evolution device (Horiba, Ltd., Kyoto, Japan). The erythrocyte sedimentation rate (ESR) was measured with a VacuplusESR-120 Fully Automated ESR analyzer, and the CRP level was measured with a Siemens Dade Behring BN II Nephelometer.

### Measurement of serum NGAL levels

Serum NGAL levels were measured by ELISA using human lipocalin-2/NGAL ELISA kits (BioVendor^®^, Brno, Czech Rep.). Diluted serum samples were added to the plates coated with NGAL antibodies. The inter-assay value (CV) was less than 10 % while the intra-assay value was 5 %. After the initial incubation for 4 h at room temperature, secondary antibody was added and incubated for an additional hour. The reaction was stopped by adding 1 M sulfuric acid. Absorbances were measured at 450 nm. The NGAL concentrations in each sample were determined from a standard curve (Mishra et al. 2005). The results were interpreted double-blindly by an investigator who did not know the patients’ diagnoses.

### Statistical analysis

IBM SPSS 21^®^ statistical software was used for statistical analysis. The initial NGAL values, Alvarado appendicitis scores, CRP, WBC count and percentage, and ESR were compared between the groups by both ROC curves and Tukey HSD tests. The NGAL values of Group 1 patients taken prior to the surgery, 24 h after the surgery and at the time of discharge were compared using the Tukey HSD test. The area under the ROC curve was calculated using SPSS software. The best cut-off point was chosen by comparing sensitivity and specificity at various levels. A p value of less than 0.05 was the criterion of statistical significance.

## Results

The average age of the study population (all groups) was 10.4 (6–16) years. Ten patients from Group 1 who underwent surgery had acute appendicitis, nine had phlegmonous appendicitis and one had perforated appendicitis. All those types of appendicitis were confirmed by a pathologist using surgically removed specimens. Five patients from Group 2 had acute gastroenteritis, four had mesenteric lymphadenitis, four had cystitis, three had constipation, and one had acute gastritis, while three patients were diagnosed with nonspecific abdominal pain. Patients from Group 3 had no infectious or inflammatorily-mediated pathologies.

**Fig. 1:**
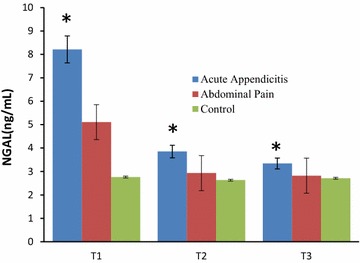
Serum NGAL levels at basal (T1), 24 h (T2) and 72 h (T3) in patients with acute appendicitis, those with abdominal pain, and controls. Serum NGAL values were significantly higher (*p < 0.05) between acute appendicitis and abdominal pain; between acute appendicitis and control; between abdominal pain and control

The mean NGAL values obtained at admission (basal) were: 8.6 ng/ml in Group 1, 3.9 ng/ml in Group 2 and 3.3 ng/ml in Group 3. The differences were statistically significant (p < 0.05) (Fig. 1). The mean NGAL values obtained at 24 h were: 5.1 ng/ml in Group 1, 2.9 ng/ml in Group 2 and 2.8 ng/ml in Group 3. Those obtained at 72 h were: 2.8 ng/ml in Group 1, 2.6 ng/ml in Group 2 and 2.7 ng/ml in Group 3.

Although the preoperative and postoperative NGAL values were significantly different (p < 0.05), there was no significant difference between the NGAL values at 24 h postoperative and at discharge (p > 0.05) (Fig. 1).

The mean serum NGAL values of the appendicitis patients from Group 1 were the highest. Those of patients with abdominal pain from the second group were lower than the first group but significantly higher than the third (control) group. There were significant correlations between Group 1 and the other groups only in terms of CRP, WBC, neutrophil percentage and ESR. There was no such correlation between Groups 2 and 3 (Table 1). In the acute appendicitis patients there was a positive correlation between NGAL level and neutrophil count (*r* = 0.408, *p* = 0.074). There were also correlations in the control group between NGAL and WBC count (*r* = 0.308, *p* = 0.187), and NGAL and neutrophil percentage (*r* = 0.481, *p* = 0.032).

**Table 1:** Alteration of CRP, WBC count, neutrophil percentage and ESR in the groups

	Group 1	Group 2	Group 3
CRP (mg/dl)	7.1^a,b^	<3^c^	<3
WBC(K/μl)	15.9^a, b^	1.5^c^	6.8
Neutrophils (%)	88.2^a, b^	59.9^c^	57.0
Sedimentation rate (mm/h)	24.0^a, b^	12.4^c^	12.2

CRP, WBC count, neutrophil percentage and ESR were significantly higher (^a^p < 0.05) in ^a^p < 0.05 between groups 1 and 2

^b^ p < 0.05 between groups 1 and 3

^c^ p < 0.05 between groups 2 and 3

**Fig. 2:**
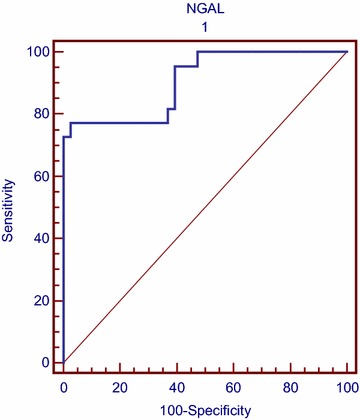
The sensitivity and specificity percentages of preoperative serum NGAL for detecting appendicitis in children. The area under the ROC curve, NGAL sensitivity of 77.3 % and specificity of 97.4 %, were identified when the Alvarado score was set to a cut-off point of “7”

When the Alvarado scores were compared with the serum NGAL levels, we found that setting the cut-off point to 7 resulted in an area under the ROC curve giving, at 95 % confidence intervals, 77.3 % sensitivity and 97.4 % specificity. With the cut-off value set to 9, the sensitivity was 100 % and the specificity 55 % (Fig. 2). On the other hand, at a cutoff value of 0.13 mg/dl for serum CRP value sensitivity was 90 %, and the specificity 65 %.

## Discussion

Abdominal pain in children is one of the most common reasons they are brought to emergency services. The correct identification of appendicitis depends on a methodical differential diagnosis and patient assessment. If the condition is not identified early and accurately, patients are at risk for perforation and development of peritonitis and/or an abscess. To diagnose appendicitis, laboratory findings to support a clinical diagnosis could be valuable. General practitioners and pediatricians serving in the emergency services can find it difficult to diagnose appendicitis without laboratory tests owing to limited clinical experience (Saraç et al. 2013). Computed tomography has a high radiation risk so it is not suited to frequent use, although it has high sensitivity and specificity on appendicitis. Therefore, in order to help in diagnosing appendicitis, scoring systems have been developed. The Alvarado scoring system was developed in 1986 and is still used in diagnosing appendicitis (Alvarado 1986), but when used alone it might not be reliable. Appendicitis-like abdominal pain in children can be confused with signs and symptoms of many other diseases. The differential diagnosis of diseases such as acute gastroenteritis, constipation, urinary tract infections, mesenteric lymphadenitis and pelvic inflammatory disease might not be straightforward (Lau et el. 1987; Rothrock et al. 1991). However, since there is no single reliable diagnostic method, diagnosis is attempted by interpreting findings from more than one parameter.

Despite all the advances, with many laboratory studies conducted to achieve a better diagnosis, appendicitis is still a significant cause of morbidity and even mortality. Although many studies have focused on 5-HIAA, CRP, procalcitonin, interleukins, leptin and NUCB2/nesfatin-1, lipopolysaccharide binding protein, etc., appendicitis is still difficult to diagnose at an early stage with such laboratory tests (Rodriguez-Sanjuan et al. 1999; Apak et al. 2005; Mentesa et al. 2009; Brănescu et al. 2012; Bakal et al. 2015). Further research is needed to develop laboratory diagnostic methods to reduce negative laparotomy rates and the incidence of perforated appendicitis.

Measurement of specific metabolites or an enzyme in serum can serve as identifiers of inflammation in specific organs. NGAL is a 25 kDa protein from the lipocalin family, which is secreted from inflammatory cells and tissues as a result of neutrophil activation (Al-Ismaili et al. 2011; Petrovic et al. 2013). In particular, it serves as an inflammatory biomarker with levels elevated in kidney and liver disorders, tumors and inflammatory diseases of the colon. Tissue NGAL levels are also reportedly increased in appendicitis; therefore, in our study, we hypothesized that serum NGAL levels are increased in appendicitis cases (Nielsen et al. 1996; Cowland and Borregaard 1997).

In this study, NGAL assays enabled us to distinguish clinically between patients who underwent appendectomy and those with nonspecific abdominal pain who had an appendicitis-like pathology and were being closely monitored owing to suspected appendicitis. Although immunohistochemical staining studies have demonstrated an increased presence of NGAL at the tissue level in appendicitis, no studies have investigated serum NGAL levels in appendicitis. Serum NGAL levels can increase for different reasons; however, we have shown here that in appendicitis patients whose diagnosis was confirmed histopathologically, serum NGAL levels are increased secondary to appendicitis.

The higher NGAL levels in patients with non-appendicitis-related abdominal pain than the control group could be attributed to temporary inflammation of the colonic epithelium. However, the mean NGAL value in the non-specific abdominal pain group was about half the value observed in the appendicitis group; though twice as high as the control group. The difference between the two groups was statistically significant.

Parameters such as CRP, WBC count, neutrophil percentage and ESR that are used to define appendicitis were significantly higher in Group 1 than the other two groups, but those values were not adequate for distinguishing between Groups 2 and 3. Unlike the above-mentioned parameters, the difference in NGAL values was significant among all three groups. Serum NGAL levels in the appendicitis group were three times higher than in the control group and this difference was statistically significant.

When serum NGAL levels in the appendicitis group were compared there was a significant difference between preoperative levels and the levels measured 24 h postoperatively and at discharge, but there was no significant difference between the 24 h postoperative and discharge values. The NGAL levels after appendectomy decreased to the level of the control group, which suggests that the elevation was due to appendicitis.

The NGAL levels were compared with the Alvarado score, setting the cut-off value to 7, which estimated the diagnosis of appendicitis an area under the ROC curve, with a 95 % confidence interval, to have 77.3 % sensitivity and 97.4 % specificity. At this cut-off point the positive predictive value for appendicitis was 94 %, while the negative predictive value was 88 %.

## Conclusions

Therefore, on the basis of our data, it seems that NGAL can be used as a laboratory parameter to differentiate appendicitis from nonspecific abdominal pain. However, in order to determine normal limit values for NGAL, a large clinical study with appendicitis or non- appendicitis patients is needed.
